# Expression and polymorphisms of T cell immunoglobulin domain and mucin domain protein-1 in thymoma with or without myasthenia gravis

**DOI:** 10.3892/ol.2014.2090

**Published:** 2014-04-25

**Authors:** KAI ZHENG, GUOWU XU, XING LU, JUN ZHANG, PENG ZHANG

**Affiliations:** Department of Cardiothoracic Surgery, Tianjin Medical University General Hospital, Heping, Tianjin 300052, P.R. China

**Keywords:** Tim-1, expression, polymorphism, thymoma, myasthenia gravis

## Abstract

The present study aimed to investigate the expression and association of the single-nucleotide polymorphism (SNP) -1637A/G in the promoter region of the T cell immunoglobulin domain and mucin domain protein-1 (Tim-1) gene in patients diagnosed with thymoma with or without myasthenia gravis (MG). The expression of Tim-1 was detected using the streptavidin peroxidase immunohistochemical staining method on tissues obtained from thymoma patients with (n=58) and without (n=62) MG. The Tim-1 gene -1637A/G polymorphism was detected using the single allele-specific primer polymerase chain reaction. The positive rate of Tim-1 expression in thymoma patients with MG was 62.1% (32/58), which was significantly higher compared with that in thymoma patients without MG (33.9%, 21/62) (P=0.002). The genotype frequencies of GG, GA and AA in the -1637A/G polymorphism were 0.7931, 0.2069 and 0, respectively, in thymoma patients with MG, and 0.6129, 0.3871 and 0, respectively, in thymoma patients without MG. A significant difference in the genotypes between the thymoma patients with MG and those without MG was found (P=0.031). In addition, a significant difference in allele frequencies between thymoma patients with MG and those without MG (P=0.024) was observed. The high expression of Tim-1 in thymoma tissues may play an important role in the development of thymoma with MG. The -1637A/G polymorphism site of the promoter region in Tim-1 may be associated with thymoma with MG. These findings provide a basis for further genetic research of thymoma with MG.

## Introduction

Thymomas are primary tumors that arise from thymic epithelial cells (TEC) (1). The thymus is a primary lymphoid organ that plays a role in regulating the proliferation and differentiation of T cells. Although the thymus typically starts to coalesce and becomes completely atrophic with remnant adipose tissue by the late teens, lymphopoiesis of the T cells continues during adult life (2). Thymomas retain thymic cortical epithelial function to induce T-cell differentiation (3); however, they may lack normal mechanisms for selection of the T cell repertoire. Autoreactive T cells possibly emerging in a thymoma may trigger autoimmune disorders (4). Thymomas are well-known for their significant association with multiple autoimmune diseases, particularly myasthenia gravis (MG). It has been reported that up to 50% of thymoma patients develop MG (5).

MG is a prototypical antibody-mediated autoimmune disease characterized by the production of autoantibodies against the skeletal muscle acetylcholine receptor (AChR) at the neuromuscular junction (6). An increasing number of muscle autoantibodies, such as muscle-specific tyrosine kinase, titin and ryanodine receptor (RyR) antibodies, have been found in patients with MG (7). MG is paraneoplastic in association with thymoma, which is detected in 10–15% of MG patients (8). Histologically, thymomas are epithelial neoplastic cells surrounded by maturing T cells. The epithelial cells are capable of expressing epitopes cross-reactive with skeletal muscle proteins, such as AChR, titin and RyR (9). The muscle-like epitopes are presented to T cells together with costimulatory molecules (9). Autoreactive T cells that are specific for AChR and titin are found in the sera of thymoma patients and thymoma patients with MG (10). Thus, autoreactive T cells play a vital role in the incidence of thymoma and MG.

The T-cell immunoglobulin domain and mucin domain (TIM) family of genes, positionally cloned in 2001 from within the T cell and airway phenotype regulator (Tapr) locus (11), consists of three members (Tim-1, -3 and -4) on the human chromosome 5q33.2 (12). TIM proteins are involved in the regulation of T helper (Th) cell immune responses and thus are key regulators of immune responses (13,14). The Th cells are subdivided into Th1 or Th2 cells based on the cytokines produced and distinct functions performed (15). The Th1 and Th2 cells play critical roles in the regulation of cellular and humoral immune responses. The balance of Th1 and Th2 cells is crucial in the immune response to several organ-specific autoimmune diseases. Tim-1, the first member of the TIM gene family, which is tightly linked to the immune system, plays an important role in the generation and/or maintenance of the balance between Th1 and Th2 cells, and is upregulated in Th2 cells following activation and interacts with its ligand expressed on antigen-presenting cells (16). It has been reported that Tim-1 polymorphisms are associated with various immune-related diseases, including rheumatoid arthritis (17), systemic lupus erythematosus (18), multiple sclerosis (19), diabetes (20), tumors (21,22) and asthma (23). However, the association of Tim-1 gene polymorphisms with thymoma and MG has not yet been studied, although it has been reported that thymoma is a tumor of the thymus, the primate lymphoid organ of T cells, and MG is an autoimmune disorder closely associated with an imbalance of Th1 and Th2 (24). The present study aimed to investigate the expression of Tim-1 in thymoma patients with and without MG and to examine whether the single-nucleotide polymorphism (SNP) -1637A/G in the promoter region of the Tim-1 gene contributes to the susceptibility of thymoma with MG. The study was approved by the ethics committee of Tianjin Medical University General Hospital (Tianjin, China).

## Materials and methods

### Reagents

Mouse anti-human Tim-1 monoclonal antibodies (mAbs), manufactured by Abcam Corporation, were purchased from Indole Biological Technology Co., Ltd (Shanghai, China). A streptavidin peroxidase (SP) test agent box was purchased from Gene Technology Co., Ltd. (Shanghai, China).

### Patients

The thymoma tissues were obtained from patients at the Tianjin Medical University General Hospital (Heping, China) from January, 2007 to April, 2013. All the samples were obtained from individuals from Northern China who were diagnosed with thymoma by clinical pathological examination. There were 58 cases of thymoma with MG, including 28 males and 30 females (mean age, 47.3 years), and 62 cases of thymoma without MG, including 38 males and 24 females (mean age, 52.7 years). Blood samples were collected from the patients in the two groups. Informed consent was obtained from all participants.

### Immunohistochemical staining

The SP immunohistochemical staining method was used according to the manufacturer’s instructions. Serial paraffin sections (five slices, 4 μm thick) were prepared. One slice was stained with hematoxylin and eosin (H&E) and the others were used for immunohistochemical analyses. The paraffin pretreatment involved xylene and alcohol graded hydration. The preparations were incubated in a 3% H_2_O_2_ for 10 min to allow endogenous peroxidase activity and microwave repair of the antigen was performed. Following three washes with phosphate-buffered saline (PBS), the samples were blocked with 5% goat serum (Shanghai Vita Chemical Reagent Co., Ltd., Shanghai, China) in PBS for 2 h at room temperature, and then incubated overnight at 4°C after adding the primary antibodies. The following day, the samples were warmed for 30 min and washed with PBS thrice for 5 min each. The samples were then incubated with the secondary goat anti-mouse polyclonal antibody (Abcam, Shanghai, China) for 30 min at room temperature, washed with PBS thrice for 5 min each, stained with diaminobenzidine for 5–10 min, rinsed with distilled water, stained with H&E, dehydrated and rinsed with xylene for 5 min. The mounting process was divided into the following two steps: i) a few drops of acacia were added to orient the samples and ii) the samples were then fixed and drops of acacia were added before covering the samples with a slide. The slides were visualized under a light microscope (XSP148AT; Shanghai Taiyi Medical Apparatus Equipment Co., Ltd., Shanghai, China). Three randomly selected fields were obtained from each slide to obtain a mean value (optical density − mean value of immunostaining intensity).

### Determining positive results

In a clear background, cells whose cytoplasm was a clear brown-yellow color or had brown granules were considered as positive cells. Cell counting was performed under a light microscope (magnification, ×400) and expressed in powers of 10. The ratio of positive to negative cells accounted for the total number of cells in a slice. The results were divided into three grades as follows: i) negative (−), no clear positive cells or <10% positive cells; ii) positive (+), 10–50% positive cells; and iii) strongly positive (++), >50% positive cells.

### SNP analysis

The genomic DNA of leukocytes from peripheral blood was extracted using sodium dodecyl sulfate lysis and proteinase K digestion (both Shanghai Vita Chemical Reagent Co., Ltd.), followed by a standard phenol-chloroform (Shanghai Vita Chemical Reagent Co., Ltd.) extraction method (25). Single allele-specific primer polymerase chain reaction was performed on 1637A/G in the promoter region of the TIM-1 gene. Primers used for polymerase chain reaction (PCR) to amplify the -1637A/G SNP in the promoter region of the TIM-1 gene are shown in Table I (OMIM: 606518) (http://omim.org/entry/606518). Wherein, allele A fragment was amplified with forward (F) 1 and reverse (R), and allele G fragment was amplified with F2 and R. The length of amplified products was 452 bp. PCR was performed with F1 and R and with F2 and R, respectively, in each sample. Touchdown PCR (Beijing AuGCT DNA-Syn Biotechnology Co. Ltd., Beijing, China) was performed in a 25-μl total reaction volume, including 1.0 μl DNA template, 2.5 μl 10× Advantage PCR buffer, 1.5 μl dNTP (2.5 mmol/l), 1.0 μl F1/F2 (5 μmol/l), 1.0 μl R (5 μmol/L), 0.5 μl DNA polymerase (2.5 U/μl) and 17.5 μl ddH_2_O. The PCR conditions were as follows: Denaturing step (95°C for 5 min), 27 cycles of chain reaction (94°C for 30 sec, annealing temperature was decreased by 1°C from 65°C to 57°C every three cycles, 72°C for 30 sec) and a final extension (72°C for 10 min). The PCR products were then detected with 1.2% agarose gel (Shanghai Vita Chemical Reagent Co., Ltd.,) electrophoresis. The PCR-amplified products of alleles G and A were directly sequenced by Beijing AuGCT DNA-Syn Biotechnology Co. Ltd.

### Statistical analysis

The χ^2^ test performed using SPSS software, version 17.0 (SPSS, Inc., Chicago, IL, USA) tested for deviation from the Hardy-Weinberg equilibrium and compared the frequency of discrete variables among thymoma patients with and without MG. P<0.05 was considered to indicate a statistically significant difference.

## Results

### Positive expression and -1637A/G polymorphism of Tim-1 in thymoma patients with MG

**Figure 1:**
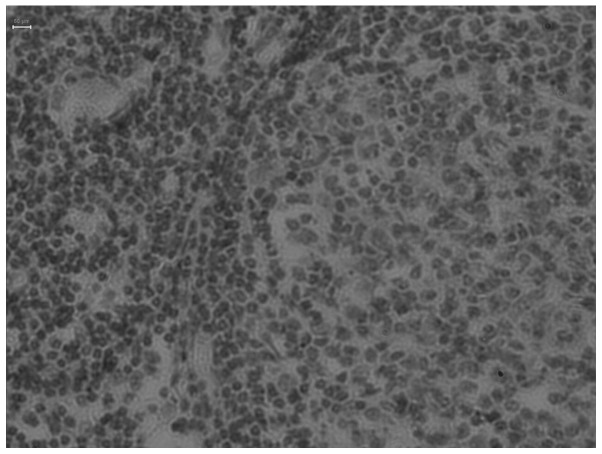
Representative image of T cell immunoglobulin domain and mucin domain protein-1 expression in thymoma tissue of a patient with thymoma in combination with myasthenia gravis.

**Figure 2:**
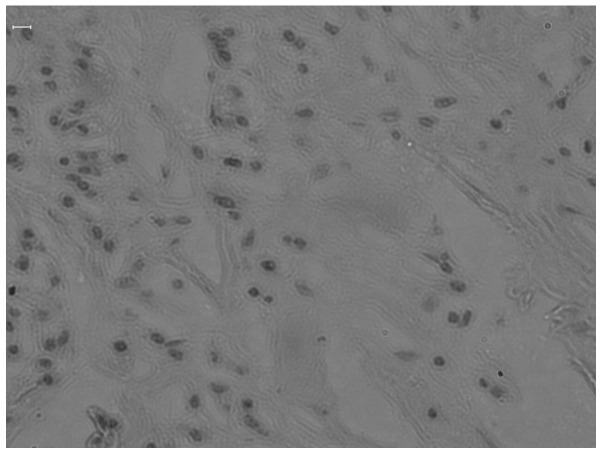
Representative image of T cell immunoglobulin domain and mucin domain protein-1 expression in thymoma tissue of a patient with thymoma without myasthenia gravis.

The expression of Tim-1 in thymoma patients with and without MG is shown in Figs. 1 and 2. Positive Tim-1 expression was determined by the presence of brown particles in the cytoplasm. The detailed data of the positive rate of Tim-1 expression are presented in Table II. The positive rate of Tim-1 expression in the thymoma with MG was significantly higher compared with thymoma patients without MG (P=0.002). The difference between the two groups was statistically significant (P<0.05).

### GG and GA genotypes and the PCR products of alleles G and A

**Figure 3:**
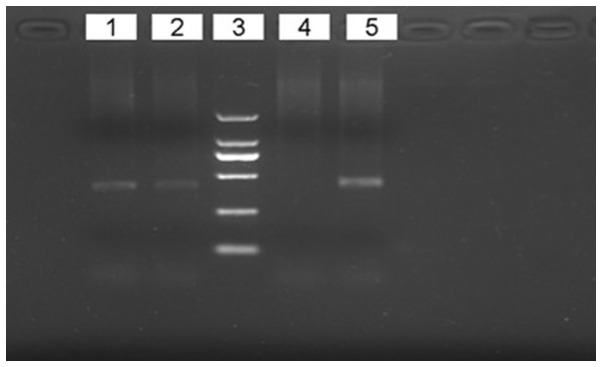
Genotype analysis of the -1637A/G SNP site of the T cell immunoglobulin domain and mucin domain protein-1 gene (1 and 2, the genotype GA; 3, DNA marker DL2000: 2000, 1000, 750, 500, 250 and 100 bp; 4 and 5, the genotype GG).

**Figure 4:**
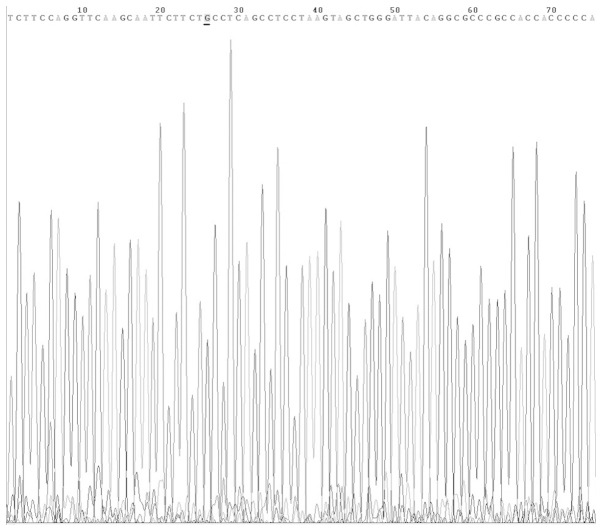
Sequence diagram of allele G amplified product at the site of the -1637 loci of the T cell immunoglobulin domain and mucin domain protein-1 promoter region. The underline for the allele G.

**Figure 5:**
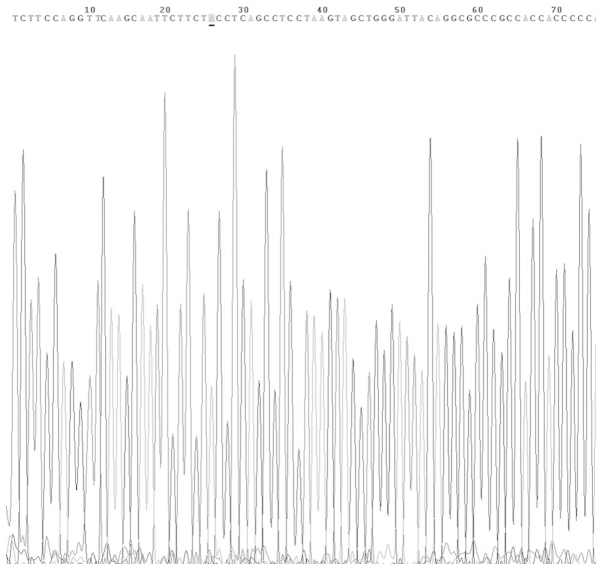
Sequence diagram of allele A amplified product at the site of the -1637 loci of the T cell immunoglobulin domain and mucin domain protein-1 promoter region. The underline for the allele A.

The GG and GA genotypes, without AA, were detected at the site of -1637A/G of the Tim-1 gene in all the cases. The results of genotype analysis are shown in Fig. 3. The PCR-amplified products of alleles G and A were sequenced and confirmed by Beijing AUGCT DNA-SYN Biotechnology Co., Ltd. (Beijing, China) (Figs. 4 and 5). In the thymoma with MG group, the G and A allele frequencies were 84.50 and 15.50%, respectively and 93.55 and 6.45%, respectively, in the thymoma without MG group.

### Positive polymorphisms of -1637A/G loci in Tim-1 with thymoma with MG

The -1637A/G polymorphism in the promoter region of Tim-1 was analyzed in thymoma patients with and without MG, and in the normal thymus group. The genotype and allele frequency distribution of the -1637A/G loci of the Tim-1 promoter region was confirmed according to the Hardy-Weinberg equilibrium principal, which suggested that the gene frequency of Tim-1 met the genetic equilibrium and was fully representative. At the -1637A/G loci, genotypes GG>GA, but not AA, were observed in all the detected samples. Additionally, the genotype frequencies at the -1637A/G polymorphic site were significantly different between thymoma patients with and without MG (P=0.031). The allele frequencies at the -1637A/G polymorphic site were significantly different between thymoma patients with and without MG (P=0.024) (Table III).

## Discussion

The TIM gene family has received significant attention since it was positionally cloned in 2001 from within the Tapr locus as a novel allergy and asthma susceptibility gene (11). Tim-1, the first family member, was initially identified in 1996 as the receptor for the hepatitis A virus (HAVCR1) in monkeys (26) and then in humans in 1998 (27). Subsequently, Tim-1 was identified as a kidney injury molecule (KIM-1) in 1998 (28). Tim-1, akin to all of the TIMs, posses a similar structure to Type 1 membrane proteins consisting of an N-terminal Cys-rich immunoglobulin variable-like domain, a mucin-like domain, a transmembrane domain and an intracellular tail. The intracellular structure of Tim-1 contains tyrosine phosphorylation motifs that are involved in transmembrane signaling (29). The signaling pathways triggered downstream of Tim-1 cross-linking have been investigated using either Tim-1 antibodies or Tim-4 as ligands. Reporter assays have shown that the overexpression of Tim-1 resulted in increased transcription from the interleukin (IL)-4 promoter and nuclear factor of activated T-cells/activator protein-1 transcriptional activation, dependent on Y276 in the Tim-1 cytoplasmic tail (30). Capping experiments using human Jurkat T cells that expressed Tim-1 suggest that Tim-1 is associated with cluster of differentiation (CD) 3 and is recruited to the T cell receptor (TCR) signaling complex in human T cells (31). In addition, this study showed that engagement of Tim-1 with agonistic Tim-1 mAbs resulted in rapid tyrosine phosphorylation of Tim-1, phosphorylation of ζ-chain-associated protein kinase 70 and IL-2-inducible T-cell kinase (ITK), as well as the recruitment of an ITK and phosphoinositide-3 kinase complex to the TCR signaling complex (31). Tim-1 is primarily expressed in activated CD4^+^ T cells (11), Th2 cells (19), at a low level on mast cells (32) and a subpopulation of B cells (33), whereas Tim-3, but not Tim-1, is expressed in Th1 cells (19). The selectively positive expression of Tim-1 between Th1/Th2 suggests that Tim-1 may be involved in diseases of immune deviation or Th1/Th2 imbalance, such as MG. The expression of Tim-1 in tumors (21,22) and the potential association between Tim-1 and thymoma, which was previously confirmed using a Tim-4-Ig fusion protein that showed the activation of T cells with Tim-4-Ig contributed to the phosphorylation of Tim-1 and thymoma viral proto-oncogene 1, indicated that Tim-1 may be involved in thymoma. These data suggest that Tim-1 may play a vital role in thymoma and MG, and formed the basis for the present study.

Previous studies have aimed to determine whether Tim-1 gene polymorphisms were associated with the incidence of asthma (11), rheumatoid arthritis (13) and hepatitis A virus infection (34). However, the association of Tim-1 with thymoma and MG has not been studied in the literature to date. To the best of our knowledge, this study is the first to investigate the expression of Tim-1 in thymoma patients with and without MG, and to examine whether the -1637A/G SNP in the promoter region of Tim-1 contributes to the susceptibility of thymoma with MG. The positive rate of Tim-1 expression in thymoma patients with MG was significantly higher compared with that of thymoma patients without MG. The genotype frequencies at the -1637A/G polymorphic site were significantly different between thymoma patients with and without MG (P=0.031). In addition, the allele frequencies at the -1637A/G polymorphic site were significantly different between thymoma patients with and without MG (P=0.024). These data suggest that Tim-1 may play a role in the development of thymoma and MG, particularly the development of thymoma with MG. However, the exact pathogenesis remains unclear. The effects of Tim-1 polymorphism on transcription and translation, and whether Tim-1 is involved in thymoma with MG via the TCR signaling pathway, requires further investigation.

In conclusion, this study demonstrated that the expression of Tim-1 in thymoma patients with MG is positive and the -1637A/G polymorphism in the promoter region of the Tim-1 gene is a potential genetic variant for the susceptibility of thymoma with MG. Further genetic studies are required to clarify the specific mechanisms involved.

## Figures and Tables

**Table I:** Primers used for amplifying the -1637A/G SNP in the promoter region of the Tim-1 gene.

SNP	Primer sequences
-1637A/G	F1: 5′-CTTCCAGGTTCAAGCAATTCTTCTA-3′ F2: 5′-CTTCCAGGTTCAAGCAATTCTTCTG-3′ R: 5′-AATCGGGCTGTTGACTTCTGCT-3′

SNP, single-nucleotide polymorphism; Tim-1, T cell immunoglobulin domain and mucin domain protein-1; F, forward; R, reverse.

**Table II:** Tim-1 expression levels in thymoma patients with MG and thymoma patients without MG.

	Tim-1 expression level (%)			
			
Thymoma patients	+/++	−	χ^2^	P-value
With MG	36	22	9.555	0.002
Without MG	21	42		

Tim-1, T cell immunoglobulin domain and mucin domain protein-1; MG, myasthenia gravis.

**Table III:** Genotype and allele analyses of the -1637A/G loci polymorphism of the Tim-1 gene promoter region.

Genotype/allele	Thymoma with MG, n (%)	Thymoma without MG, n (%)	P-value
GG	46 (79.31)	38 (61.29)	0.031
GA	12 (20.69)	24 (38.71)	-
AA	0	0	-
G	98 (84.50)	116 (93.55)	0.024
A	18 (15.50)	8 (6.45)	-

Tim-1, T cell immunoglobulin domain and mucin domain protein-1; MG, myasthenia gravis.
